# Leptomeningeal carcinomatosis in a patient with recurrent unresectable squamous cell carcinoma of the retromolar trigone—a brief report

**DOI:** 10.1186/s43046-022-00147-y

**Published:** 2022-11-07

**Authors:** Jhansi Pattanaik, Varshu Goel, Priyanka Sehrawat, Ruchi Rathore, Rajesh Kumar Singh, Ajay Garg, Ahitagni Biswas

**Affiliations:** 1 Department of Radiotherapy & Oncology, All India Institute of Medical Sciences, New Delhi, 110029 India; 2 Department of Neurology, All India Institute of Medical Sciences, New Delhi, India; 3 Department of Pathology, All India Institute of Medical Sciences, New Delhi, India; 4 Department of Neuroradiology, All India Institute of Medical Sciences, New Delhi, India

**Keywords:** Leptomeningeal carcinomatosis, Carcinomatous meningitis, Head and neck squamous cell carcinoma, Radiotherapy, Methotrexate

## Abstract

**Background:**

The reported incidence of leptomeningeal carcinomatosis is 3–8% in patients with solid tumours. More commonly, it has been described in the setting of advanced cancers of the lung, breast and malignant melanoma.

**Case presentation:**

A 50-year-old diabetic patient with recurrent unresectable squamous cell carcinoma (SCC) of the right retromolar trigone (rT4bN0M0) presented with severe low backache and weakness in bilateral lower limbs 20 days after the completion of concurrent chemoradiotherapy. Contrast-enhanced MRI of the spine showed multiple nodular enhancing leptomeningeal lesions at the lumbar level and an intramedullary T2/FLAIR-hyperintense longitudinal lesion involving the central cord from C2 to C7 vertebral levels, suggestive of leptomeningeal metastases. Cerebrospinal fluid (CSF) analysis revealed pleocytosis, elevated protein and markedly decreased glucose. The CSF cytology revealed scattered large atypical cells, suspicious for metastasis. Non-contrast MRI of the brain showed a T2/FLAIR-hyperintense lesion involving the right caudate nucleus suggestive of either an acute infarct with haemorrhagic transformation or a haemorrhagic brain metastasis. During assessment, he had high-grade fever and was started on empirical intravenous antibiotics (ceftriaxone, vancomycin and subsequently meropenem) in line with the management for acute bacterial meningitis. Gram staining of CSF did not demonstrate the presence of any bacteria and the specimen was sterile on culture. He did not respond to empirical antibiotics, had a progressive downhill course and eventually died due to aspiration pneumonia.

**Conclusion:**

This brief report highlights the importance of awareness of leptomeningeal carcinomatosis as a possible cause of backache with sensorimotor deficit and autonomic dysfunction in a previously treated case of head and neck SCC.

## Background

The common causes of severe low backache and paraparesis in a patient with cancer include metastasis to the spinal cord, the vertebrae or the lumbosacral plexus. In this context another probable cause is carcinomatous meningitis, also known as leptomeningeal carcinomatosis, which being a rare entity, is difficult to diagnose unless there is high suspicion for the same. Leptomeningeal carcinomatosis has been reported in less than 10% of patients with solid tumours, more commonly in the setting of advanced cancers of the lung, breast and malignant melanoma [1–3]. We herein describe a case of carcinomatous meningitis in a patient with recurrent unresectable squamous cell carcinoma (SCC) of the retromolar trigone and illustrate the key clinical and laboratory findings in support of the diagnosis.

## Illustrative case

A 50-year-old north Indian type 2 diabetic male presented to the emergency department with complaints of acute pain in the abdomen, constipation, severe low backache radiating to bilateral lower limbs, weakness and inability to walk without support for the past 10 days. Two years prior to these events, he was diagnosed with well-differentiated SCC of the right retromolar trigone cT2N0M0, for which he underwent wide local excision of the tumour with segmental mandibulectomy, upper alveolectomy and modified radical neck dissection type III. The postoperative histopathology report revealed a pT2N0 tumour with close (0.4 cm) superolateral margin. He did not opt for post-operative radiation therapy and was lost to follow-up during the first wave of the COVID-19 pandemic and the consequent nation-wide lockdown. He presented to our centre 18 months after surgery with complaints of progressively increasing trismus and local pain at the operated site. On further evaluation, including a whole body 18F-FDG positron emission tomography/computed tomography (PET/CT) scan, a 5.2 × 4.2 cm FDG-avid soft tissue mass in the tumour bed with lytic lesions in the mandible, suggestive of local recurrence (rT4bN0M0), was detected. In view of high infratemporal fossa involvement, salvage re-surgery was ruled out and he was subsequently planned for concurrent chemoradiotherapy to a dose of 65 Gray to the high-risk planning target volume (PTV) and 54 Gray to the low-risk PTV (bilateral neck nodal levels Ia, Ib, II, III and right-sided level IVa, Va and Vb) by simultaneous integrated boost-volumetric modulated arc therapy (SIB-VMAT) technique in 30 fractions over 6 weeks along with concurrent cisplatin at a dose of 40 mg/m^2^ weekly. Twenty days after the completion of concurrent chemoradiotherapy, he presented with the aforementioned complaints to the emergency department.

**Fig. 1:**
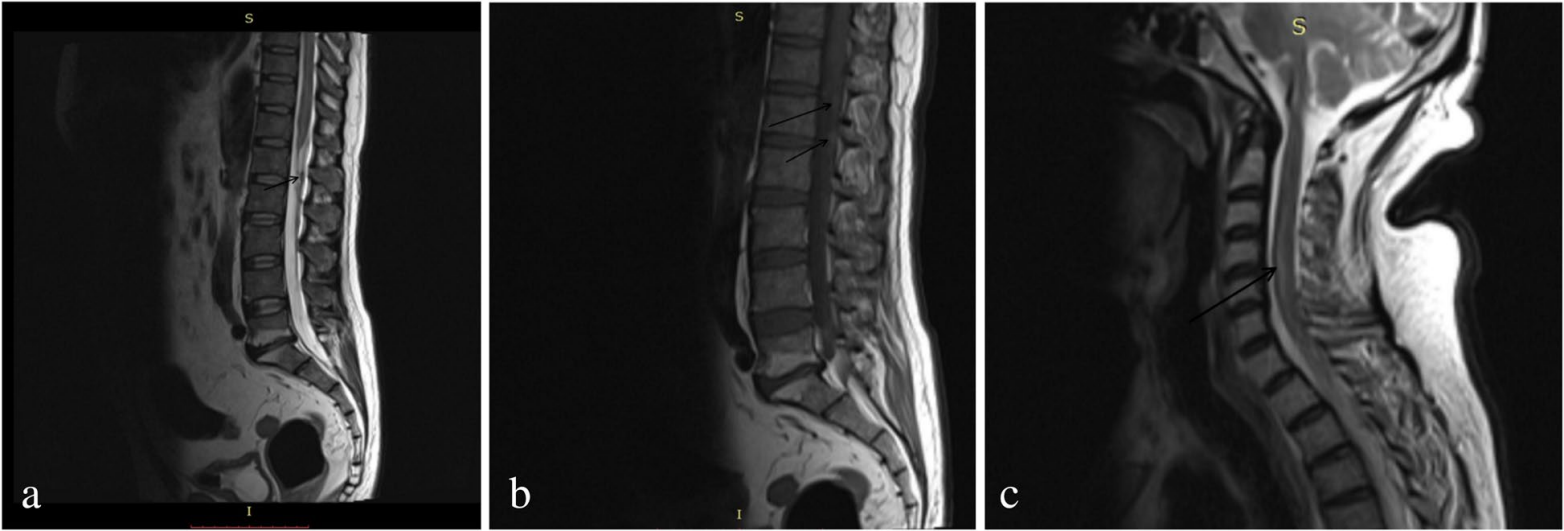
T2-weighted (**a**) and post-contrast T1-weighted (**b**) sagittal MRI of the lumbosacral spine showing nodular leptomeningeal lesions at lumbar level (L1), signal hyperintensities in the endplates of L5 and S1 with disc bulge at L4–L5 and L5–S1 levels causing thecal sac indentation; T2-weighted MRI of the cervical spine (**c**) showing an intramedullary hyperintense longitudinal lesion involving the central cord from C2 to C7 vertebral level, suggestive of leptomeningeal carcinomatosis

On physical examination, he was afebrile and haemodynamically stable. On neurological examination, he had decreased tone in the lower limbs bilaterally. The power was MRC grade 2 in the right lower limb and grade 1 in the left lower limb. There was decreased sensation below the level of the umbilicus (T10 spinal segment) and loss of bladder and bowel control. After eliminating sub-acute intestinal obstruction as a cause of abdominal pain and constipation, a contrast-enhanced magnetic resonance imaging (MRI) of the lumbo-sacral spine along with a screening of the whole spine was done to rule out compressive myelopathy and the patient was prophylactically started on injection dexamethasone 8 mg TDS. The MRI scan revealed decreased intervertebral disc space at L5–S1 and disc bulge at L4–L5 and L5–S1 levels causing thecal sac indentation and compression of the exiting nerve roots (Fig. 1). Also, thecal sac indentation was noted at C4–C5 and C5–C6 levels due to prolapsed intervertebral disc (PIVD). In addition, an intramedullary T2/FLAIR-hyperintense longitudinal lesion involving the central cord from C2 to C7 vertebral levels and multiple nodular enhancing lesions along the leptomeninges of the spinal canal at the lumbar level were noted suggestive of leptomeningeal metastases (Fig. 1). A neurosurgical opinion was sought for the management of PIVD but any need for active intervention was ruled out. A contrast-enhanced computed tomography (CT) scan of the head and neck revealed hypodense foci in the right frontal lobe and the right basal ganglia, suggestive of infarcts and a stable primary tumour. He was started on aspirin and atorvastatin in order to prevent further episodes of cerebrovascular accident.

**Fig. 2:**
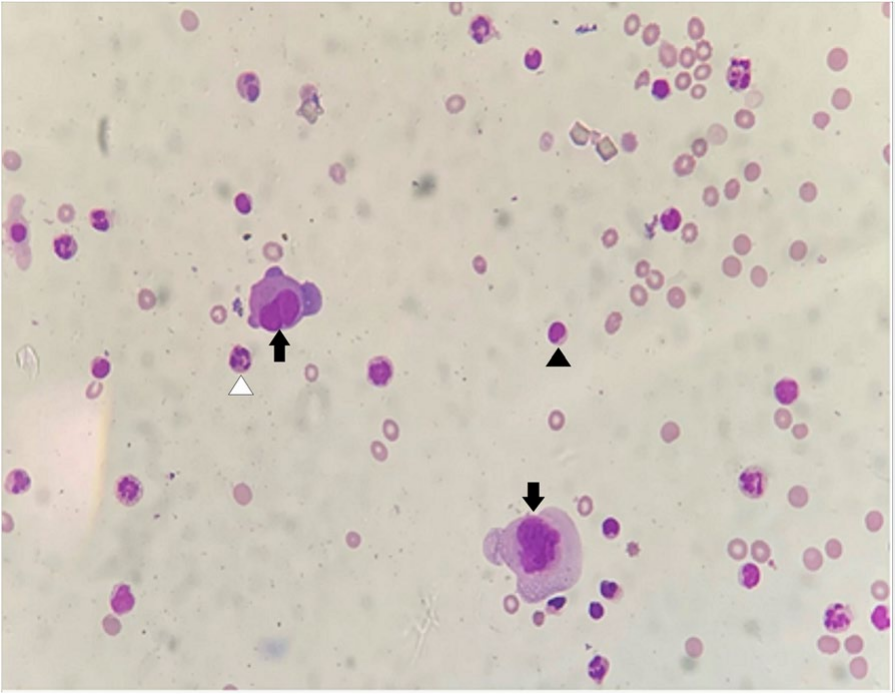
Cerebrospinal fluid cytology showing scattered large atypical cells (black arrows) in the background of lymphocytes (black arrowhead) and neutrophils (white arrowhead)

**Fig. 3:**
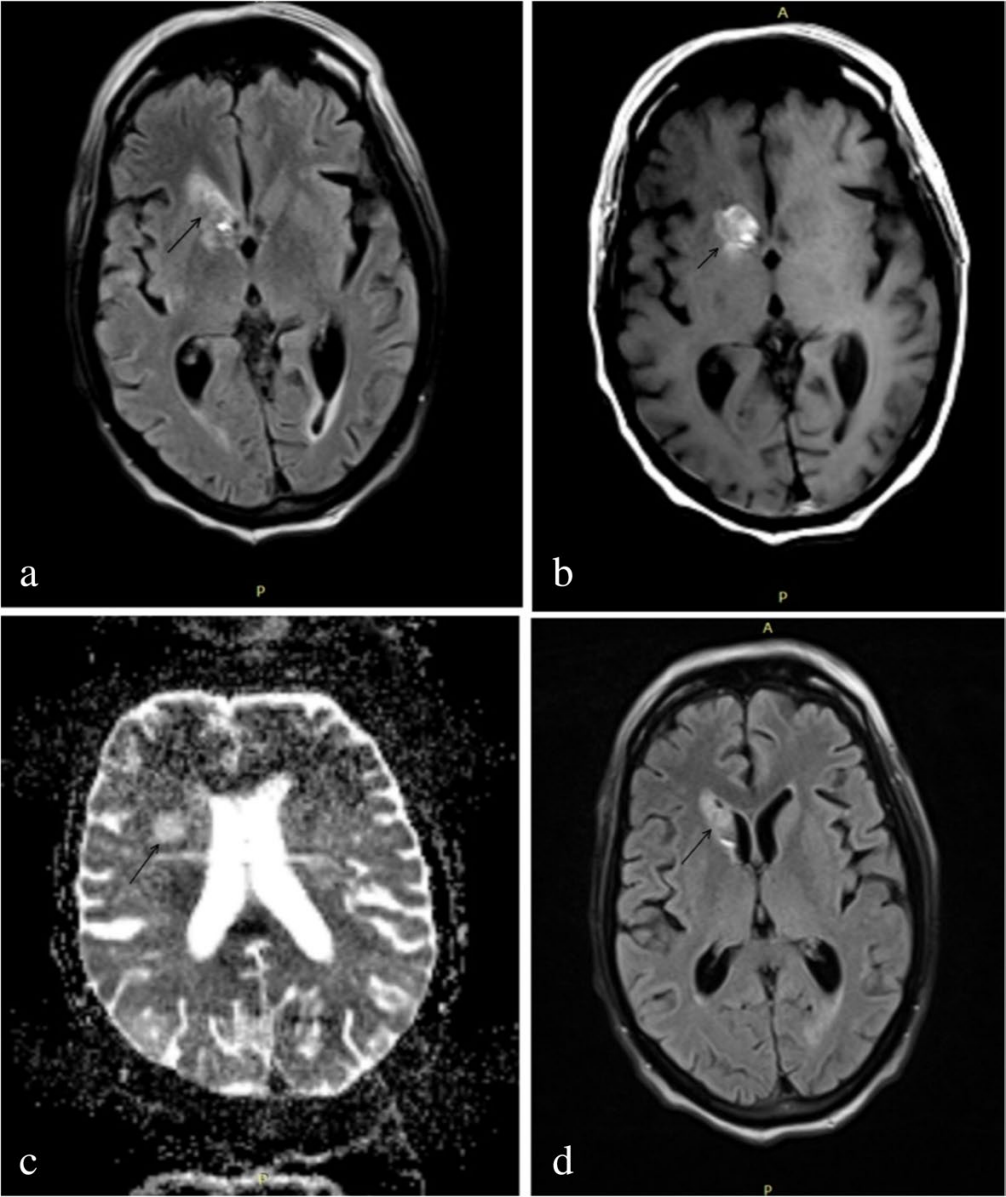
MRI of the brain showing a hyperintense lesion involving the right caudate nucleus on FLAIR (**a**, **b**), diffusion-weighted (**c**) and non-contrast T1-weighted (**d**) axial images suggestive of an infarct with haemorrhagic transformation or haemorrhagic metastais to the brain

Meanwhile, he had two episodes of high-grade fever and a lumbar puncture was performed. Cerebrospinal fluid (CSF) analysis revealed an elevated cell count (30/mm^3^, 70% neutrophils), elevated protein (788 mg/dl) and markedly decreased glucose levels (32 mg/dl; corresponding serum glucose levels—168 mg/dl). He was started on empirical intravenous (IV) antibiotics (ceftriaxone, vancomycin and subsequently meropenem) in line with the management for acute bacterial meningitis. However, Gram staining of CSF did not demonstrate the presence of any bacteria and the specimen was sterile on culture. It was negative for both cryptococcal antigen testing and cartridge-based nucleic acid amplification test (CBNAAT) for tuberculosis. The CSF cytology revealed scattered large atypical cells, which were suspicious for metastasis (Fig. 2). He subsequently underwent a non-contrast MRI of the brain which revealed a T2/FLAIR-hyperintense lesion involving the right caudate nucleus suggestive of either an acute infarct with haemorrhagic transformation or a haemorrhagic metastasis to the brain (Fig. 3).

In view of a high index of suspicion of carcinomatous meningitis and no clinical improvement on IV antibiotics, a lumbar puncture was repeated after four days. Analysis of CSF revealed markedly elevated protein (777 mg/dl) and low glucose levels (19 mg/dl; corresponding serum glucose levels—179 mg/dl). The repeat CSF cytology showed numerous polymorphs and occasional lymphonuclear cells with no evidence of atypical metastatic cells. Meanwhile, he had neurological deterioration and developed quadriparesis with lower motor neuron (LMN) type bilateral VIIth cranial nerve and left-sided IIIrd cranial nerve palsies. A repeat contrast-enhanced MRI of the brain and CSF cytology were planned for confirmation of the diagnosis but he became dyspnoeic due to aspiration pneumonia and was electively tracheostomized. He was put on mechanical ventilation but subsequently deteriorated and eventually succumbed to his illness.

## Discussion

Carcinomatous meningitis is defined as an infiltration of the leptomeninges (the arachnoid membrane and the pia mater) by malignant cells. This condition, synonymous with ‘leptomeningeal carcinomatosis’ (LCM) and ‘meningeal carcinomatosis’, is an uncommon manifestation of solid malignancies [1–3]. Eberth is credited with the first published observation of leptomeningeal metastases in lung cancer in 1870 [4]. The multifocality of signs and symptoms is attributable to multiple tumour deposits throughout the neuraxis. Tumour cells enter this neuraxial space through multiple mechanisms such as direct extension from parenchymal disease through the pia mater, via arterial and venous channels by haematogenous spread, extension from bone either directly or through veins, seeding from subependymal disease, spillage into CSF cavities from surgery and retrograde invasion along peripheral nerves or their lymphatics [5–7]. The diagnosis of LCM is associated with poor prognosis with a reported median overall survival (OS) of 2.3–4.7 months in modern series [8].

Carcinomatous meningitis is estimated to occur in approximately 3 to 8% of patients with solid tumours [1, 3]. The solid tumours most often associated with leptomeningeal spread are carcinoma of the breast, lung, and malignant melanoma [1, 2, 7, 9]. In up to 10–20% of cases, symptoms secondary to carcinomatous meningitis may precede the diagnosis of the primary tumour [10, 11]. Common symptoms include headache, a change in the mental status, back or radicular pain, nausea, vomiting, limb weakness, sensory complaints, diplopia, dysphagia, dysarthria, and incoordination. Signs may include alteration in the mental status, cranial nerve palsies, asymmetry of deep tendon reflexes, lower motor neuron weakness, and sensory deficits [2–7]. Seizures may occur owing to infiltration of the brain parenchyma [2]. Of note, nuchal rigidity is present in only 7–17% of cases and fever is typically absent [1, 2, 7–9]. According to a review by Gleissner and Chamberlain, spinal symptoms (>60%) are the most common, followed by cerebral (50%) and cranial nerve symptoms (40%) in patients with LCM [12].

The diagnosis is often difficult to establish despite strong clinical suspicion. Conventionally, the establishment of a definitive diagnosis requires the finding of malignant cells in the CSF on cytological examination, but at least 3 lumbar punctures may be required to ascertain the diagnosis. A single CSF examination reveals positive findings in approximately 50% of patients and this percentage rises to 85 to 90% after 3 procedures [7, 9]. Cytological results remain negative in some patients despite repeated lumbar punctures. These false-negative findings may result from the strong adherence of malignant cells to the leptomeninges or due to the presence of focal rather than widespread leptomeningeal tumour [9]. Other CSF markers such as elevated protein levels, raised cell count, low glucose concentration, raised opening pressure and elevated levels of tumour markers may give an indication of the presence of LCM [7, 9]. In the illustrative case, the CSF analysis showed increased protein concentration, low glucose, and an elevated white cell count (predominantly neutrophils). The first CSF cytology revealed scattered large atypical cells, suggestive of leptomeningeal metastases.

Contrast-enhanced MRI is the radiographic modality of choice for the diagnosis of LCM. The entire neuraxis must be imaged as multifocal involvement is common. In most patients, the MRI will reveal leptomeningeal enhancement that is frequently associated with cranial nerve enhancement and gross tumour deposits [11]. It may additionally include contrast enhancement of the sulci, basilar cisterns, cauda equina and hydrocephalus [3]. The sensitivity of Gadolinium (Gd) enhanced MRI is equivalent to that of CSF analysis. However, the specificity of Gd-MRI (77%) is lower than that of the CSF examination (100%). Hence, MRI could be of diagnostic value, especially when CSF cytology is negative. In such an instance, it can differentiate between patients with a low or high risk of LCM. Nevertheless, a negative MRI following negative CSF does not exclude LCM [13].

There is no consensus on the optimal management of patients with LCM. This is mainly because of the lack of large published experiences, limited number of randomized trials, nonuniform treatment regimens in single institution experiences and inclusion of various primary tumour histologies in the clinical trials. However, an aggressive central nervous system (CNS) directed treatment plan comprising radiation therapy (whole brain radiotherapy and/or focal spinal radiation to symptomatic sites) and intrathecal chemotherapy (methotrexate, cytarabine or thiotepa) should be considered in patients with good performance status. The commonly used palliative radiotherapy regimens are 20 Gray in 5 fractions over 1 week and 30 Gray in 10 fractions over 2 weeks with the latter being preferred in patients with relatively more favourable prognosis. Craniospinal irradiation to the entire neuraxis may be considered in select patients with diffuse leptomeningeal metastases. Usually, CNS-directed therapy is given in conjunction with tumour-specific systemic therapy in fit patients. In this context, drugs that cross the blood-brain barrier (intravenous high dose methotrexate, ifosfamide, thiotepa and oral temozolomide, capecitabine, small molecule tyrosine kinase inhibitors, e.g. gefitinib, erlotinib and lapatinib) may be considered depending upon tumour sensitivity. The incorporation of novel biological agents targeting individual tumour-specific mutation in the systemic and CNS-directed therapy is an innovative approach. Despite the aforementioned treatment approaches, the overall prognosis remains poor. Without any treatment, the OS is approximately 6 weeks, and with appropriate treatment, the median OS increases to approximately 3–6 months [1, 3, 9, 11]. The patient, discussed in this report, had Eastern Cooperative Oncology Group (ECOG) performance status (PS) 3 at presentation which rapidly evolved to ECOG PS 4, which precluded the use of anticancer treatment. The option of intrathecal methotrexate monotherapy after the planned 3rd lumbar puncture was discussed with the patient’s relatives but the patient succumbed to his illness before the procedure.

Head and neck squamous cell carcinomas (HNSCC) are considered curable malignancies, if diagnosed in the early stage. In locally advanced HNSCC, patterns of failure are usually local, regional or locoregional. Though distant metastases are relatively rare, they are difficult to cure. The reported incidence of distant metastases in HNSCC varies widely in the published literature and is usually between 10 and 25% [14, 15]. The most common site of distant metastases is the lungs, accounting for half to two-thirds of all distant metastases, followed by bones and liver [14, 15]. Leptomeningeal metastases from HNSCC have been scarcely described in the medical literature (Table 1) [16–22]. In the context of HNSCC, LCM has been mostly reported in cancers involving the lip, paranasal sinus and nasopharynx, due to the propensity for perineural spread and intracranial extension through the cribriform plate and skull base foramina [16, 17, 20–22]. To our knowledge, we have described the first case of carcinomatous meningitis in a patient with SCC of the retromolar trigone. Retrograde perineural spread of cancer cells along the mandibular (V3) nerve from the recurrent tumour in the post-op bed, right masticator space and infratemporal fossa could represent a likely pathway of LCM in this patient.

**Table 1:** Compendium of cases of leptomeningeal carcinomatosis (LCM) from primary head and neck squamous cell carcinoma

Author, year of publication	Age (years)/sex	Primary cancer site, histology and initial management	Time to LCM	Presenting symptoms of LCM	Imaging findings	CSF analysis	Treatment of LCM	Survival outcome
Banerjee et al. [16] (1984)	70/male	Carcinoma lip (SCC); wide local excision of tumour	8 months	Right mental neuropathy, right 5th, 7th, 8th CNP, complete ophthalmoplegia	CT head—normal. There was biopsy-proven metastases to mandible	Protein—elevated, glucose—decreased, cytology—positive for tumour cells	Weekly intravenous MTX and bleomycin	Died after 12 months from the diagnosis of LCM and 26 months from the diagnosis of cancer
Redman et al. [17] (1986)	30/male	Carcinoma of ethmoid sinus (SCC); Chemotherapy: CDDP, VCR, Bleomycin f/b RT	7 months	Nausea, vomiting, decreased vision in left eye, decreased sensation over the left side of face, status epilepticus	CT scan—normal	Protein—elevated, cytology- positive for tumour cells	Whole brain RT➔ weekly IT MTX	Died after 10 months from the diagnosis of LCM
Redman et al. [17] (1986)	29/male	Carcinoma nasopharynx (PDSCC); surgery ➔ RT; recurrence in oropharynx after 6 months: surgery ➔ RT	4 months	Bifrontal headache with nausea, vomiting, dizziness; optic atrophy on right side and papillitis on left side; anisocoria, 6^th^ cranial nerve palsy (6 months after diagnosis of LCM); seizure (10 months after diagnosis of LCM)	Initial CT scan normal; CT scan at 2 months after diagnosis of LCM—bifrontal extradural tumour extension with involvement of both optic nerves; CT scan at 7 months after diagnosis of LCM—bifrontal meningeal enhancement with no intracranial lesion	At 7 months after diagnosis of LCM: protein—elevated, glucose—normal, cytology—no malignant cell; At 10 months after diagnosis of LCM: protein—elevated, glucose—decreased, cytology—positive for tumour cells	Systemic chemotherapy—CDDP and 5FU	Died after 11 months from the diagnosis of LCM
Redman et al. [17] (1986)	34/male	Carcinoma base of tongue with cervical lymph node metastasis (SCC); surgery ➔ RT	16 months	Occipital headache with blurred vision in left eye; blindness in the left eye, decreased vision in the right eye (3 months after diagnosis of LCM); motor seizure (6 months after diagnosis of LCM)	CT scan—tumour recurrence with extension to left orbital apex, cavernous sinus and sphenoid sinus	Protein—elevated, cytology—negative for tumour cells; At 8 and 13 months after diagnosis of LCM: same as before; CSF from Ommaya reservoir positive for malignant cells	Systemic chemotherapy—CDDP and 5FU; weekly intraventricular chemotherapy with MTX via Ommaya reservoir	Died after 22 months from the diagnosis of LCM
Redman et al. [17] (1986)	44/male	Carcinoma of right ethmoid sinus (PDSCC)	At presentation	Anosmia, cephalgia, right-sided proptosis, anisocoria, right lateral gaze palsy	CT scan—obliteration of ethmoid sinus with an extension of tumour through the floor of the anterior fossa, compression of the right lateral ventricle	Protein—elevated, cytology—positive for tumour cells	Systemic chemotherapy-CDDP and 5FU; steroids and emergency cranial decompression; cranial RT; weekly intraventricular chemotherapy with MTX	Died after 21 months from the diagnosis of LCM
Biswal et al. [18] (1998)	50/male	Carcinoma right tonsil cT3N2aM0 (WDSCC); Palliative RT (30 Gy/10 fr/2 weeks) ➔ radical conversion to equivalent dose of 70 Gy	7 months	Headache, deafness, diplopia, nasal regurgitation, hoarseness, bilateral 7th and 8th cranial nerve palsies	MRI—normal, Gd DTPA scan—prepontine deposit; biopsy proven metastatic skin nodules; CXR—multiple lung metastases	CSF cytology-positive for malignant cells	Palliative RT to whole brain 20 Gy/5 fr/1 week	Died after 1 month from the diagnosis of LCM
Thompson et al. [19] (2003)	51/male	Carcinoma left glottis T1N0M0 (SCC); Definitive RT (60 Gy/30 fr); Carcinoma left supraglottic larynx (SPC after 12 years) T1N3M0 (SCC): laryngectomy and MRND ➔ adjuvant RT to right neck (50 Gy/25 fr) and RT to left neck (60 Gy/30 fr)	5 months	Low backache, decreased sensation in both lower limbs, constipation, inability to bear weight	MRI lumbar spine—leptomeningeal enhancement; CXR—normal	CSF cytology-atypical cells	IT MTX RT(20 Gy/5 fr) to T12- S3 level	Died after 3 weeks from the completion of palliative RT
Lee et al. [ 20] (2005)	43/female	Carcinoma nasopharynx (SCC); RT and concurrent chemotherapy with cisplatin and 5FU	4 years	Right-sided facial weakness, numbness, loss of taste and smell, pain in right maxillary area, twitching in right facial musculature, right retroorbital pain, diplopia	MRI at 2 years after diagnosis of LCM: recurrent nasopharyngeal mass with involvement of ethmoid sinus and extension to frontotemporal leptomeninges	CSF cytology—negative for malignant cells	Systemic chemotherapy—MTX, 5FU with leucovorin	Died after 3 years from the initial diagnosis of LCM
Sullivan et al. [21] 2006	51/male	Carcinoma lower lip(SCC); Local excision of tumour; Local recurrence after 3 years (SCC); wide local excision of tumour with mandibular resection➔ post-op RT (54 Gy/27 fr)	4 years	Paraesthesia over right cheek and forehead Right radicular pain, right foot drop, left leg paraesthesia, decreased sensation in L4-L5 dermatomes on right and S1, 2 dermatomes on left (during stereotactic RT to the right cavernous sinus lesion)	Ill-defined enhancing mass below right foramen ovale with abnormal enhancement and thickening in cavernous sinus and PNI of the right trigeminal nerve MRI spine during stereotactic RT to right cavernous sinus lesion—multiple meningeal nodules in cervical and lumbar spine	Protein—elevated, cytology—negative for malignant cells	Stereotactic RT to the right cavernous sinus lesion (66 Gy/33 fr) Dexamethasone 4 mg QID and whole spinal RT (35 Gy/15 fr)	Died after 3 months from the completion of spinal RT
Pougnet et al. [22] (2014)	33/male	Carcinoma lip (WDSCC) post resection with perineural invasion of the trigeminal nerve within temporal fossa; Definitive CTRT (70 Gy/35 fr) with concurrent cisplatin and 5FU	12 months	Paraparesis and back pain Progressive back pain, sphincter disorder and lower limb weakness (24 months after diagnosis of LCM)	Medullary myelitis without meningeal enhancement; MRI at 27 months after diagnosis of LCM—meningeal nodule and leptomeningeal enhancement	CSF cytology—no atypical cells At 27 months after diagnosis of LCM: protein—elevated, glucose—decreased CSF cytology—atypical cells consistent with metastatic SCC	Dexamethasone; dysimmune myelitis-Mycophenolate mofetil At 24 months after diagnosis of LCM: weekly ITMTX and MPS+ systemic chemotherapy with 3 weekly carboplatin and weekly cetuximab	Patient was alive at 6 months of the start of treatment of IV and IT chemotherapy

*SCC* squamous cell carcinoma, *CSF* cerebrospinal fluid, *CT* computed tomography, *RT* radiotherapy, *VCR* vincristine, *5FU* 5-fluorouracil, *CDDP* cisplatin, *MTX* methotrexate, *LCM* leptomeningeal carcinomatosis, *ITMTX* intrathecal methotrexate, *PD* poorly differentiated, *WD* well-differentiated, *SPC* second primary cancer, *CTRT* concurrent chemoradiotherapy, *CXR* chest X-ray, *Gd DTPA* gadolinium: diethylenetriamine pentaacetic acid, *MRI* magnetic resonance imaging, *Gy* Gray, *BOT* base of tongue, *fr* fractions, *MRND* modified radical neck dissection, *WLE* wide local excision, *CNP* cranial nerve palsy, *MPS* methyl prednisolone, *IV* intravenous, *IT* intrathecal

## Conclusion

Although locoregional failure is the most common pattern of failure in HNSCC in general, the incidence of distant metastasis is slowly increasing due to more effective locoregional disease control with the advancement of multimodal treatment strategies including surgery, radiotherapy and chemotherapy as well as improvement in diagnostic imaging. In the context of backache with sensorimotor deficit and autonomic dysfunction in a previously treated case of HNSCC, compressive myelopathy due to spinal metastasis is an important differential diagnosis. However, this brief report underpins the importance of awareness of leptomeningeal carcinomatosis as another likely possibility in this setting. Despite the grave prognosis and limited survival, early diagnosis of LCM may provide the cancer physicians a window to offer CNS-directed treatment, e.g. cranial or spinal RT and intrathecal chemotherapy for the palliation of symptoms and improvement of health-related quality of life.

## Data Availability

All data generated or analysed during this study are included in this published article.

## Abbreviations

5FU: 5-Fluorouracil
CBNAAT: Cartridge-based nucleic acid amplification test
CDDP: Cis-diamine-dichloro-platinum
CNP: Cranial nerve palsy
CNS: Central nervous system
CSF: Cerebrospinal fluid
CT: Computed tomography
CTRT: Concurrent chemoradiotherapy
CXR: Chest X-ray
DTPA: Diethylenetriamine pentaacetate
ECOG: Eastern Cooperative Oncology Group
FDG: Fluro deoxy glucose
FLAIR: Fluid-attenuated inversion recovery
Gd: Gadolinium
Gy: Gray
HNSCC: Head and neck squamous cell carcinoma
IT: Intrathecal
IV: Intravenous
LCM: Leptomeningeal carcinomatosis
LMN: Lower motor neuron
MPS: Methyl prednisolone
MRC: Medical Research Council
MRI: Magnetic resonance imaging
MRND: Modified radical neck dissection
MTX: Methotrexate
PDSCC: Poorly differentiated squamous cell carcinoma
PET: Positron emission tomography
PIVD: Prolapsed intervertebral disc
PS: Performance status
PTV: Planning target volume
RT: Radiotherapy
SCC: Squamous cell carcinoma
SIB-VMAT: Simultaneous integrated boost-volumetric modulated arc therapy
TDS: Three times a day
VCR: Vincristine
WDSCC: Well-differentiated squamous cell carcinoma
WLE: Wide local excision
